# Post-Transfusion Purpura: A Case Report of an Underdiagnosed Phenomenon

**DOI:** 10.7759/cureus.1207

**Published:** 2017-05-01

**Authors:** Hind Rafei, Raza Yunus, Samah Nassereddine

**Affiliations:** 1 Department of Internal Medicine, The George Washington University

**Keywords:** post-transfusion purpura, transfusion reaction, thrombocytopenia, platelet count

## Abstract

Post-transfusion purpura is a rare transfusion-related complication that often goes undiagnosed. It is due to alloimmunization against platelet antigens which leads to acute profound thrombocytopenia following the transfusion of any platelet-containing product (red blood cells or platelets). It is commonly seen in multiparous women. Here, we report a case of post-transfusion purpura in a 56-year-old multiparous woman who developed acute thrombocytopenia seven days following a packed red blood cell transfusion. We will discuss the clinical presentation, diagnosis, workup and treatment of this rare disease. It is important to recognize this entity separately and to include it in the differential diagnosis of acute thrombocytopenia after a recent blood transfusion. Treatment for this condition consists of intravenous immunoglobulins, corticosteroids or plasmapheresis.

## Introduction

Post-transfusion purpura (PTP) is a rare and delayed transfusion reaction that typically occurs in multiparous women. It occurs after transfusion of any platelet-containing product (red blood cells or platelets) causing acute profound thrombocytopenia [1]. It is caused by alloimmunization against platelet antigens, anti-human platelet antigen-1a (HPA-1a) being the most frequent antibody involved [2]. The diagnosis is made by clinical suspicion combined with serological ﬁndings. The presence of alloantibodies to known platelet antigens and the lack of these antigens on the patient’s platelets is suggestive of PTP [3]. It is important recognize this entity separately in order to treat appropriately. We report a case of PTP and discuss the clinical presentation, diagnosis, and management of this rare condition.

## Case presentation

**Figure 1:**
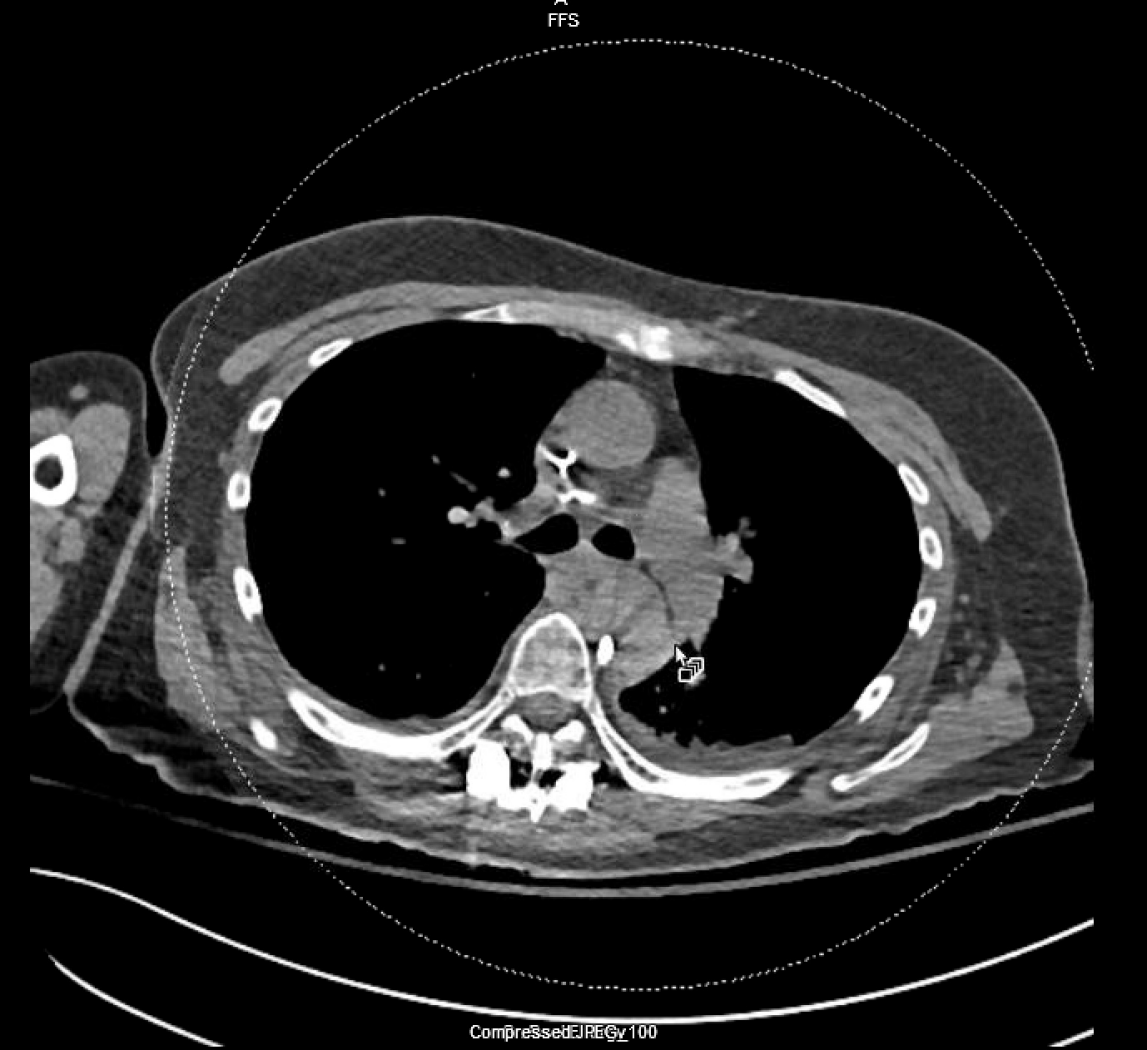
Computed tomography (CT) scan of the chest An aortic intramural hematoma is demonstrated with screw abutting the proximal descending thoracic aorta.

A 56-year-old multiparous woman with a past medical history of hypertension, gastroesophageal reﬂux disease (GERD), chronic obstructive pulmonary disease (COPD) and scoliosis presented for elective revision of the spinal hardware. On postoperative day 1, she developed atypical chest pain. A chest computed tomography scan revealed an aortic intramural hematoma with screw abutting the proximal descending thoracic aorta (Figure 1).

She was transferred to the intensive care unit (ICU). She underwent thoracic endovascular aortic repair (TEVAR) with aortic graft stent placement. She received 5,000 units of unfractionated heparin during this procedure and three units of packed red blood cells after the procedure. The day following the procedure, the ICU team started the patient on low-molecular-weight heparin for deep venous thrombosis prophylaxis. Seven days after the procedure, her complete blood counts showed an acute drop in platelet count from 193,000/mcL to 49,000/mcL in 24 hours. On vital signs, she had a heart rate in the 90s. She required three liters of oxygen by nasal cannula to maintain adequate oxygen saturation. On the physical exam, she had diffuse wheezes over all lung ﬁelds. Her skin exam was signiﬁcant for scattered ecchymoses over her bilateral upper extremities with no petechiae. Her complete blood count revealed a platelet count of 49,000/mcL and a hemoglobin level of 9.3 gm/dL. The day prior, her platelet count was 193,000/mcL and her hemoglobin level was 10.7 gm/dL. At the time of the acute drop in the platelet count, the kidney function and electrolytes were within normal limits. Her liver function tests were signiﬁcant for an elevated aspartate aminotransferase (AST) to 84 units/L and alanine aminotransferase (ALT) to 96 units/L. Coagulation studies were as follows: international randomized ratio (INR) = 1.24, prothrombin time (PT) = 15.6 seconds, partial thromboplastin time (PTT) = 36 seconds, D-dimer = 4.96 mcg/mL, and ﬁbrinogen = 718 mg/dL. Lactate dehydrogenase (LDH) was 1158 units/L (Table 1).

**Table 1:** Laboratory findings on day seven following thoracic endovascular aortic repair (TEVAR)

General hematology	
WBC	7,040/mcL (4,500-11,000/mcL)
Hemoglobin	9.3 gm/dL (12-15.5 gm/dL)
Hematocrit	28.9% (34.9-44.5%)
Platelets	49,000/mcL (150,000-450,000/mcL)
General chemistry	
Sodium	138 mEq/L (135-145 mEq/L)
Potassium	3.7 mEq/L (3.5-5 mEq/L)
Chloride	99 mEq/L (96-106 mEq/L)
Bicarbonate	29 mEq/L (23-30 mEq/L)
Blood urea nitrogen	13 mg/dL (7-20 mg/dL)
Creatinine	0.7 mg/dL (0.5-1.1 mg/dL)
Total bilirubin	0.6 mg/dL (0.3-1 mg/dL)
Direct bilirubin	0.0 mg/dL (0.1-0.3 mg/dL)
Indirect bilirubin	0.2 mg/dL (0.2-0.7 mg/dL)
Alkaline phosphatase	115 U/L (44-147 U/L)
Aspartate transaminase	84 U/L (10-40 U/L)
Alanine transaminase	96 U/L (7-56 U/L)
Lactate dehydrogenase	1,158 U/L (140-280 U/L)
Coagulation	
Prothrombin time	15.6 seconds (11-13.5 seconds)
International normalized ratio	1.24 (0.8-1.1)
Partial thromboplastin time	36 seconds (25-35 seconds)
Fibrinogen	718 mg/dL (150-400 mg/dL)
D-dimer	4.96 mcg/mL (less than 0.38 mcg/mL)

**Figure 2:**
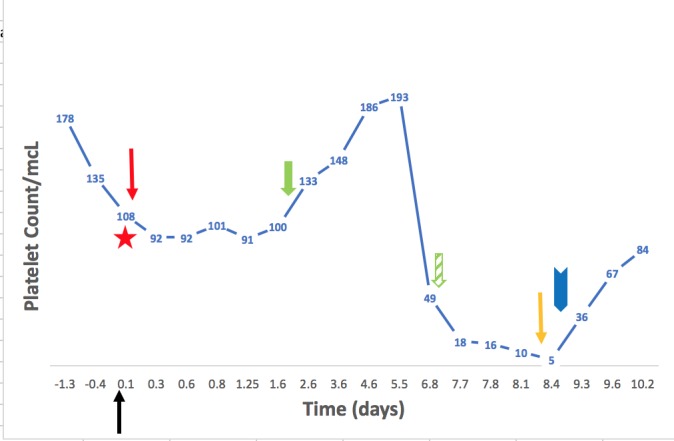
Evolution of platelet count with time Time zero corresponds to the thoracic endovascular aortic repair (TEVAR) (black arrow). The star corresponds to the administration of 5,000 units of unfractionated heparin during the procedure. The red arrow corresponds to the transfusion of three units of packed red blood cells immediately after the procedure. The solid green arrow corresponds to the initiation of low-molecular-weight heparin for deep venous thrombosis prophylaxis. The dashed green arrow corresponds to the stop of low-molecular-weight heparin. The orange arrow corresponds to the transfusion of two units of platelets. The blue arrow corresponds to the initiation of intravenous immunoglobulins therapy.

The platelet count continued to trend down until reaching 5,000/mcL over the course of the following 48 hours (Figure 2). The hemoglobin and the other laboratory studies remained stable.

The differential diagnosis included: heparin induced thrombocytopenia (HIT), PTP, disseminated intravascular coagulation (DIC), medication-induced thrombocytopenia, thrombotic thrombocytopenic purpura (TTP), and post-TEVAR thrombocytopenia. The medications were reviewed. There were no agents know to be associated with thrombocytopenia. DIC was unlikely due to the lack of other associated clinical and laboratory ﬁndings (the absence of schistocytes on peripheral smear and the absence of ﬁbrinogen consumption). Concerning post-TEVAR thrombocytopenia, the platelet count did drop immediately after the procedure from 135,000/mcL prior to the TEVAR to a nadir of 91,000/mcL in the 24 hours following the procedure. This initial drop was thought to be associated with the procedure itself. However, the platelet count recovered again to reach 193,000/mcL in six days following the procedure. Hence, the drop seven days after was thought to be less likely due to post-TEVAR thrombocytopenia. PTP was favored due to the typical timing of the platelet transfusion and onset of thrombocytopenia, as well as the severity of thrombocytopenia. Supportive care with platelet transfusion was instituted. The patient was started on intravenous immunoglobulins (IVIg) 400 mg/Kg of body weight and anticoagulation was held. The patient improved; the platelet counts increased to 84,000/mcL in 48 hours. The treatment was continued for five days. Heparin-induced antibodies as well as serotonin-release assay (SRA) results were negative. Human platelet antigen (HPA-1a) was absent suggesting that PTP is likely the underlying etiology of thrombocytopenia. The drop in the platelet count can also be explained by a passive antibody transfer from a previously sensitized donor. However, the timing from platelet transfusion to the onset of thrombocytopenia suggested that the patient might have developed HPA-1a antibodies when exposed to platelets carrying the HPA-1a antigen.

## Discussion

PTP was ﬁrst described in 1961 by Shulman, et al. [4] as profound and acute thrombocytopenia that usually occurs five to 10 days after a blood transfusion. The incidence, as reported in the literature, varies between 1:50,000 to 1:100,000 transfusions [5]. PTP occurs because of complement ﬁxation of platelets if they carry a speciﬁc antigen [4]. The antigen most commonly involved is HPA-1a [2]. Initially, the patient’s platelets that lack this antigen, get immunized when exposed to blood products containing this antigen. When exposed to blood products containing HPA-1a positive platelets again, immune complexes form and lead to thrombocytopenia. This explains the fact that this entity is more commonly seen in multiparous women who were exposed to fetal HPA-1a positive platelets [6]. Other antigens that mediate PTP have been described. HPA-1b, HPA-3a, HPA-3b and HPA-4b antibodies have all been reported in case reports either singly or in combination as the culprit antibodies [5].

The diagnosis of PTP is based on certain serologic ﬁndings. These include the presence of circulating alloantibodies to common platelet antigens and the absence of the corresponding antigens on the patient’s own platelets [3]. PTP is an immunologically-mediated phenomenon where immune complexes form leading to thrombocytopenia. The diagnosis of PTP might be challenging, especially that other immune-mediated entities leading to thrombocytopenia such as immune thrombocytopenic purpura (ITP) share similar peripheral blood smear and bone marrow aspirate and biopsy ﬁndings. A meticulous review of medications can help rule in or out drug-induced thrombocytopenia. Discontinuing a recently initiated medication that is historically associated with thrombocytopenia is essential. However, a history of blood product transfusion seven to 10 days prior to the development of thrombocytopenia should strongly suggest PTP [7]. PTP is a life-threatening condition that should be suspected early. While the serologic tests are not always readily available at the time of presentation, a high clinical suspicion should prompt immediate treatment.

Treatment of PTP includes IVIg, corticosteroids or plasmapheresis [8]. The first-line therapy is IVIg in high doses consisting of 400 to 500 mg/Kg per day usually for five days. For severe thrombocytopenia, a higher dose can be given (1 g/Kg per day) for two days. The platelet count usually starts to exceed 100,000/mcL about four days from the initiation of the treatment [9]. Platelet transfusion is generally not effective [9]. When needed, HPA-1a-negative patients should be transfused with HPA-1a-negative blood products. If not available, red blood cell washing to remove contaminating HPA-1a-positive platelets can be attempted [9]. Recurrence of PTP has been previously reported in the literature. Subsequent transfusions for patients with prior PTP should utilize an antigen-negative blood product or autologous blood [10].

## Conclusions

PTP should be on the differential diagnosis for severe thrombocytopenia that occurs about a week after blood transfusion. The typical patient is a middle-aged multiparous woman, as previous pregnancy with or without transfusion constitutes a culprit for exposure to platelet antigens. Diagnosis is conﬁrmed by the hematological ﬁndings and platelet serologies. It is crucial to correctly identify this condition as it has a particular treatment consisting of plasmapheresis, IVIg or corticosteroids.
